# Medical-legal partnerships: An integrated approach to advance health equity and improve health outcomes for people living with HIV

**DOI:** 10.3389/frph.2022.871101

**Published:** 2022-08-25

**Authors:** Omar Martinez, Miguel Munoz-Laboy, Robin Davison

**Affiliations:** ^1^College of Medicine, University of Central Florida, Orlando, FL, United States; ^2^School of Social Welfare, Stony Brook University, Stony Brook, NY, United States

**Keywords:** HIV continuum of care, medical-legal partnerships, HIV care, social service, health-harming legal needs, legal aid

## Abstract

Medical Legal Partnerships (MLPs) offer a structural integrated intervention that could facilitate improvements in medical and psychosocial outcomes among people living with HIV (PLWH). Through legal aid, MLPs can ensure that patients are able to access HIV services in a culturally sensitive environment. We conducted organizational-level qualitative research rooted in grounded theory, consisting of key informant interviews with MLP providers (*n* = 19) and members of the Scientific Collaborative Board (SCB; *n* = 4), site visits to agencies with MLPs (*n* = 3), and meetings (*n* = 4) with members of the SCB. Four common themes were identified: (1) availability and accessibility of legal and social services support suggest improvements in health outcomes for PLWH; (2) observations and experiences reveal that MLPs have a positive impact on PLWH; (3) 3 intersecting continua of care exist within MLPs: HIV care continuum; legal continuum of care; and social services continuum; and (4) engagement in care through an MLP increases patient engagement and community participation. The MLP approach as a structural intervention has the potential to alleviate barriers to HIV/AIDS treatment and care and thus dramatically improve health outcomes among PLWH.

## Introduction

HIV is embedded in social and economic inequity (1–4). Despite biomedical advances in HIV treatment, HIV continues to be a significant global public health issue with approximately 9.5 million people living with HIV (PLWH) around the world are not engaged in ART treatment (5–7). Of the nearly 1.2 million people aware of their diagnosis in 2021 in the Unites States, 81% were linked to medical care within 1 month of diagnosis, 76% had received HIV medical care, 58% were retained in care, and 66% had achieved viral suppression (8). The prevalence rate of HIV is inversely related to socio-economic status (9–12), and directly related to social determinants of health (SDoH), which are the economic and social conditions that influence individual and community health (13–16).

SDoH include a range of factors, including income, education, employment, housing, food and nutrition, insurance coverage and health care access, access to transportation, personal and family stability, and social support networks (17). Many PLWH experience barriers to health that cannot be managed with medical treatment alone; many others have no access to medical treatment at all, let alone additional supports. Research shows that these and other SDoH account for 60% of a person's health outcomes (18). SDoH affect PLWH's capacity to manage their illness and maintain their health (19, 20). For example, PLWH who are homeless or have insecure or unsafe housing are more likely to have limited access to regular medical care, to delay HIV care, and are less likely to adhere to HIV treatment (21). In many communities, PLWH are at risk of losing their housing due to factors including high medical costs, limited income, and compromised ability to work due to related illnesses (16, 21). Accessing and maintaining treatment for HIV is often complicated by co-occurring conditions, including substance use disorders, mental illness, and other structural barriers, such as incarceration (22–24).

In the early 2000s, researchers, practitioners, and policy makers began designing and implementing structural interventions to prevent HIV transmission, increase access to HIV treatment, and reduce community-level viral load. By 2015, it was evident that HIV medication adherence requires tremendous, multilevel support (25). Globally, most HIV structural interventions can be categorized as: (1) enhancing access to stable housing; (2) supporting micro-enterprises to improve financial independence; (3) improving education and job training; and (4) integrating health services, including mental health and substance use, into HIV services (26). Medical Legal Partnerships (MLPs) are rarely acknowledged in discussions of HIV structural interventions, though they have the potential to support advances in all 4 categories, to address barriers to HIV care, and to improve health outcomes.

Medical Legal Partnerships create a healthcare delivery model that integrates clinical care and legal assistance, and often includes social services (27, 28). MLPs have emerged as an innovative and effective approach to improve patient outcomes by addressing health-harming legal needs and SDoH (29–33). A health-harming legal need is a conflict between an individual and a government or private entity or a deprivation of a civil or legal right that directly or indirectly adversely affects a person's access to, or retention in, health care (34, 35). Addressing health-harming legal needs can mitigate the effects of SDoH across the life course of PLWH.

MLPs are built on three key principles: (1) the social, economic, and political context in which people live has a fundamental impact on health; (2) SDoH often result in issues that require legal assistance; and (3) attorneys are uniquely qualified to provide this legal support. According to a 2016 study by the National Center for Medical-Legal Partnership in the United States, 86% of MLPs reported improved patient health outcomes and 64% reported improved patient compliance with medical treatment (36). MLPs are based on the concept that patient health outcomes can be improved by simultaneously addressing the medical needs of PLWH and the non-medical factors that create barriers to health care and retention in treatment. By providing legal support to reduce social stressors, MLPs enable PLWH to prioritize health issues. Through a combination of legal support and services delivered through legal partners who are positioned to operate ethically with clinics and patients, MLPs can reduce health disparities. Therefore, this analysis examines MLPs as a potential structural intervention to mitigate the barriers to care and improve positive movement in the HIV care continuum. We also present the MLP approach as a structural intervention that has the potential to alleviate barriers to HIV/AIDS treatment and care and thus dramatically improve health outcomes among PLWH.

## Methods

### Design

After almost 40 years of the HIV epidemic, it is critical to take a structural approach to developing sustainable solutions (37). A structural approach recognizes that societal-level factors such as poverty, gender power relationship, social norms, social networking, and policies are critical underlying drivers of the global HIV epidemic (38). Thus, the study methodology draws on the principles of institutional ethnography and institutional case studies to examine MLP practices as the unit of analysis. Through key informant interviews, group interviews with staff of MLPs, and field site visits, organizational level qualitative data was generated. Researchers conducted interviews with 19 MLP key informants, visited three organizations involved in MLPs, and conducted four meetings with SCB members. Data were collected from 2018 to 2020. Grounded theory was used to analyze the organizational data (39).

### Sites

Based on formative work conducted the year prior to the start of the study and meetings with the leadership at the National Center for Medical Legal Partnerships, investigators selected an initial sample of 8 MLPs working with PLWH and, through referral, contacted one additional MLP. These MLPs are located in eight states and a district: Pennsylvania, New York, New Jersey, California, Hawaii, Wisconsin, Maryland, Florida and Washington, DC. Three sites visited were selected for field visits to examine three types of combination of MLP services: (a) organization provides on-site legal services; (b) organization provides referrals to outside legal services; (c) organization has a legal advocate on site and provides referrals for legal services off-site.

### Key informants

The researchers conducted interviews with 19 key informants–lawyers, health providers, social workers, administrators, and researchers–with specific expertise and experience with the MLP approach to care. When possible, they interviewed two key informants per MLP. Three site visits to existing MLPs were conducted, one meeting with all Scientific Collaborative Board (SCB) members was convened, and discussions with individual SCB members were held. The levels of experience with MLP services for PLWH ranged from 4 to 20+ years.

### Interview guide and procedures

The interview guide was designed by the authors and contained 10 questions. Sample questions included: How would you describe the MLP approach in your agency? What factors contribute to the success of the MLP and why? What are the main challenges of MLP implementation? The interviews were conducted in person or by phone, with three of the authors alternately asking questions, followed by unscripted follow-up questions. During each interview, the authors took notes independently and subsequently compared recorded responses for accuracy. Discrepancies were clarified within 1 week of the interviews through internal discussions and conversations with the interviewees.

### Scientific collaborative board

The six Scientific Collaborative Board members, consisting of four attorneys, one MLP social worker, and one clinician, met with the researchers to review and refine findings and provide guidance on next steps, including preparation for a subsequent NIH grant submission. The investigators took notes from the meetings.

### Data analysis strategy

This study employed qualitative methods rooted in the grounded theory tradition. Grounded theory is a rigorous qualitative research method in which investigators examine a situation in order to understand how key players manage their roles and then, through inductive reasoning, develop a theory or framework to convey an understanding of the situation or phenomenon (40). In this study, data collection and analysis followed the grounded theory constant comparative approach, viewing data collection and analysis as a single, concurrent process in which the method evolves as the data unfolds.

The investigators first focused on “within” differences among key informants' MLPs with regard to the provision of HIV services. After determining the organizational structure and goals, the investigators identified the various approaches to MLP HIV services and the underlying rationales. Key components of MLP structure and implementation were later validated by the SCB. Further discussions identified organizational-level practices, including practices related to screening strategies for health harming legal needs and the continua of care; structure and staffing, including staffing of legal aid providers and operational hours; communication and information sharing among MLP partners; and written informational materials for clients and providers, including content of biomedical HIV prevention marketing initiatives like Pre-Exposure Prophylaxis (PrEP) and Treatment as Prevention (TasP). The investigators used field notes to record these discussions.

## Results

Four major themes were identified (1) the availability and accessibility of ancillary legal and social services support improves health outcomes for PLWH; (2) key informants relied on their organizational philosophies and experiential anecdotes to deduce the importance of MLPs for HIV patients; (3) three intersecting continua of care that impact PLWH outcomes exist within MLPs: HIV care continuum; legal continuum of care; and social services continuum; and (4) patient engagement and community participation increases were attributed to the perceived effect of these combined continua of care.

### Organizational commitment to legal and social service support

Key informants consistently affirmed that the availability and accessibility of legal and social services support improves health outcomes for PLWH. All key informants stressed the importance of addressing SDoH and health-harming legal needs to improve health outcomes. Overall, they affirmed that providing legal support and social services on-site is integral to the delivery of HIV prevention and care. The presence of the attorney on-site enabled a more natural, fluid attorney-client relationship to develop. The patients, already comfortable in the health care environment with their health care team, felt more at ease connecting with attorneys in this setting. An attorney explained that “*having that ability to help people in a place that they trust and there's consistency and these are the people who live in their community with them, and they are working with us, that is what, it really makes a huge difference to us.”* It also provides the opportunity to engage the patient in legal services where they are, without requiring another appointment with an outside person at another location, which often creates more barriers to legal services access. Health care and social service providers felt their ability to introduce patients to attorneys at the time legal issues were discovered made it far more likely that their patients would engage in legal support. Anecdotally, this seemed to be true whether the patient was able to meet with the attorney at that moment or the appointment was scheduled for a week or two after the introduction. An attorney explained that the people most in need of legal services are also the least likely to seek it. The benefit of having an attorney in-house is that this structure provides access to people who may have limited means, opportunity, or ability to access legal support on their own. He said, “*Well we know that, by definition, if people have the wherewithal to make it to legal services offices they're in a lower risk category than a lot of the people that we serve.”* It should be noted that many of the MLPs maintained an appointment schedule for their lawyers, with time held open for walk-ins and patients with time-sensitive legal needs, such as evictions and domestic violence.

There was consensus that the optimal MLP structure is a co-location model through which medical, legal, and social services are provided within the health services organization, however this is not the only model through which MLPs can effectively operate. Many MLPs have successfully helped patients through referrals to off-site legal partners for support. Among the various challenges highlighted, regardless of model, three of the most significant are managing communication among MLP partners; educating medical providers about their critical role in meeting the legal needs of their patients; and securing stable and sufficient funding. One communication challenge specifically mentioned was the sharing of information among MLP partners without compromising attorney-client privilege or violating HIPAA regulations. With regard to funding, challenges included identifying resources within the health system to provide on-site legal services and securing funding for social service support within legal aid organizations.

### Observations and anecdotes supporting the importance of MLPs for PLWH: “*It is the right thing to do”*

Because the MLP key informants interviewed have focused on identifying and mitigating barriers to healthcare and providing high-quality medical and legal services to PLWH, evaluation efforts, including cost-benefit analysis and measurement of patient-level outcomes for those who did and did not receive MLP services, have received less attention. As one key informant explained, “We don't do it because it's good business, we do it because it's the right thing to do.” This sentiment was reflected by other informants as well, therefore they relied on their experiences and observations to reaffirm the importance of maintaining MLPs for PLWH. One MLP participant is conducting an analysis correlating health outcomes with legal intervention.

All participants expressed significant interest in measuring the impacts of the MLP approach on health outcomes and cost-benefit, specifically with regard to discussions about the emerging health care cost models which focus on population level outcomes, reduction of health care costs, and with regard to HIV, reduction of community level viral load. MLP practices voluntarily released organizational level data on outcome indicators that they trace. One MLP in particular, The Doral MLP (a pseudonym), collected cost-benefit data. The Doral MLP started in late 2016. In the first 6 month period of implementation, the Doral MLP was able to demonstrate: (a) 92% improvement in health-harming legal needs screening; and, (b) 98% improvement in patient knowledge of their rights and health-harming legal needs; using a pre-post evaluation design. Direct financial benefits were defined as reduction in debt, receipt of child support payments, food stamps, SSDI, and other payments obtained on behalf of the clients. They also reported that the crude legal intervention cost per client reduced (−37.2%) from 2017 to 2018 while maintaining the same level of average direct financial benefit to clients.

### Integrated organizational conceptualization of HIV continuum of care

All MLPs in this study provided HIV clinical care according to the HIV care continuum model, which outlines the progression of HIV medical care for PLWH, from initial diagnosis to viral suppression. The five stages of this model are: HIV diagnosis, linkage to clinical care, engagement and retention in care, receipt of ART therapy, and achievement of viral suppression.

Key informants identified the integration of legal support, social services, and clinical care as the key to improving outcomes in the HIV care continuum and achieving optimal health and wellness. They also identified numerous barriers to HIV/AIDS care. Some examples include:

1. Without health insurance coverage, provider visits and prescription medications are inaccessible.
2. Without stable housing, PLWH are far less likely to take daily medications.
3. Without accessible transportation, PLWH have difficulty traveling to medical appointments.
4. Without access to food stamps, nutrition can be compromised.
5. Without employer-provided accommodations in accordance with the Americans with Disabilities Act, the risk of unemployment is high.
6. Without education and job training, stable jobs are scarce.

Medical Legal Partnership providers stressed the need to conduct comprehensive screening for patients' legal and social services early on in the HIV care engagement. Screening methods varied among MLPs, but some methods included oral interviews, paper screenings, or patients' self-reporting. Based on the needs identified, patients were then connected to legal services and social services.

Though most of the MLPs interviewed provided at least some social service support on site, only one provided comprehensive legal support and social services on site at the location of HIV care. It is less common for legal services to be located on site at the health care organization due to several factors, including a lack of funding for lawyers on site and the availability of quality legal aid services through independent, long-standing organizations with expertise and infrastructure.

Investigators identified a legal continuum of care and social service continuum that, when implemented in conjunction with the HIV care continuum, have the potential to address health-harming legal needs, SDoH, improve health outcomes, and strengthen communities.

### Legal continuum of care

Based on the examination of legal services by MLPs, these services can be examined as a continuum of care. The qualitative organizational data collected suggest five stages of the legal continuum of care are: identification of health harming legal need(s), linkage to legal support, engagement in legal services, resolution of legal issue(s), access to continued legal support. The identification of at least one health harming legal need triggers the legal continuum of care, though key informants reported most PLWH have two or more legal issues. When a legal need has been identified and the patient expresses an interest in legal support, the patient arrives at the first step of the legal continuum–linkage to counsel. There are various models through which MLPs make legal services available, which turn on where and when the linkage occurs. Some MLPs linked individuals to on-site legal support co-located at the health center/medical center; others refer their patients to off-site legal partners. The timing of linkage to legal support may vary based on whether the legal support is available on-site or off-site, availability of appointments, and the urgency of the legal issue. The time-sensitivity of legal services is most pronounced when urgent issues arise, such as eviction, utility shut-off, and orders of protection, making immediate access (same day) to legal support is critical. Though other legal issues, such as denials of benefits or employment accommodations, might not require same-day legal intervention, it is important for connection to legal support to occur quickly. Though more research is needed to determine the optimal time frame for linkage to legal support, it appears that connection to legal services within 2 days may be most predictive of legal engagement. The key informants all agreed that a critical predictor for success of legal intervention is the time between identification of a health harming legal need and connection to legal support. The likelihood that patients will avail themselves of accessible legal services decreases with the passage of time.

Once the individual is engaged in legal support, the attorney and client discuss the client's life situation, barriers to health, and goals. Investigators detected two distinct approaches to identifying legal issues: the “screen-in” approach and the “screen-out” approach. For MLPs that screen-in, the goal is to identify and address as many legal issues as possible. For MLPs that screen-out, the organization seeks to handle a specific cadre of legal issues, often based on the more common needs of the particular patient populations they serve and the availability of legal resources/specialists to address those issues. In both situations, once the attorney-client relationship has been established, the attorney can initiate or respond to legal action on behalf of the client. Legal representation continues through resolution of the matter. Legal work may require brief engagement, such as writing a letter demanding a landlord remediate mold, helping the client file paperwork for a legal name and gender change, or appealing a decision denying benefits. There may be other instances where the legal work requires more protracted work, or even litigation. More research is needed to determine the proportion of legal intervention that falls in each category, and will likely differ based on the populations served by the MLPs.

If the matter is resolved in the client's favor, anecdotal evidence suggests that there is often an improvement in the client's SDoH, which can lead to improvements in health and economic conditions, such as reliable housing, improved access to benefits, or job training and employment opportunities. These outcomes have direct health impacts, such as improved medication adherence and viral suppression, decreased ER visits, reduced stress levels, and better mental health treatment. There may also be indirect benefits, including steady employment, family stability, and improved quality of life. Because of the individualized nature of SDoH and health harming legal needs, it is not possible to rely on one data point to analyze health outcomes for all recipients of MLP services and support, however all informants observed improvements to SDoH in the populations they serve.

Lastly, an important component of the legal continuum is the continued availability of legal support to address additional issues as they arise. As part of this continuity of legal support, some MLPs incorporate an educational component to their services. For example, lawyers from an MLP legal service provider in Florida conduct legal workshops on multiple topics to educate clients about their legal rights, empower them to become self-advocates, and maintain an ongoing relationship with the client, making it easier for individuals to access legal support in the future.

### Social services continuum of care

Social services support may be warranted if the initial patient screening identifies these needs, if a legal issue is not resolved in the client's favor, or if there is no legal resolution for the issue that was originally referred to legal services. There is a vast array of social service supports available, depending on patients' individual needs, however patients don't always know how or where to access these services. Patients may need help applying for benefits, dealing with trauma, accessing treatment for substance use, or finding mental health counseling. In these instances, the identification of the need for social service intervention triggers a referral to the appropriate social service(s). The closer in time this linkage occurs, the more likely it is that these issues will be successfully managed. When patients are engaged with case managers, peer navigators, patient educators, support groups, transportation, food, and emergency assistance, they are more likely to adhere to ART and achieve and maintain viral suppression. Ongoing access to social services support is critical to long-term management of medical, behavioral, substance use, mental health, and other issues, and to address new issues as they arise.

One key informant identified the need for legal intervention as an indicator of a breakdown in social services support, explaining that most barriers to health can be addressed early on through social services, when the issues are less complicated and less urgent. When these issues aren't effectively addressed through social services, they can quickly become crises requiring legal intervention. All of the key informants agreed on the importance of screening to identify social service needs early in the clinical engagement.

Another key informant noted the importance of capturing SDoH from patients as a precursor to identifying patients' needs for legal support and social services. This key informant noted the growing trend toward including SDoH in the medical record to develop a more complete profile of the individual and highlight potential issues.

### Sustainability and impact through community participation

Among the MLP key informants interviewed, several pointed to community participation and engagement as a way to assess the impact of the integration of care through MLPs. One key informant illustrated this point through the experience of a transgender woman who sought legal assistance for a legal name and gender change. With the support of the MLP legal team, she legally changed her name and gender (where applicable) on her birth certificate, social security card, driver's license, passport, and voter registration, deriving significant benefits to her emotional health, safety, and privacy. These experiences inspired her to become an advocate for the transgender community and she personally connected ~2,000 people to legal services for legal name and gender changes, enabling them to reap the same benefits, thereby strengthening the community. It is important to note that in this instance, through the initial connection for legal support, many of the transgender clients began receiving medical care and other services through the MLP. Another key informant explained that the ultimate success of MLPs can be measured by a person's engagement in the community and participation in the political process. Overall, key informants highlighted the rise in patient engagement in care and community participation as significant outcomes of the MLP approach.

## Discussion

**Figure 1:**
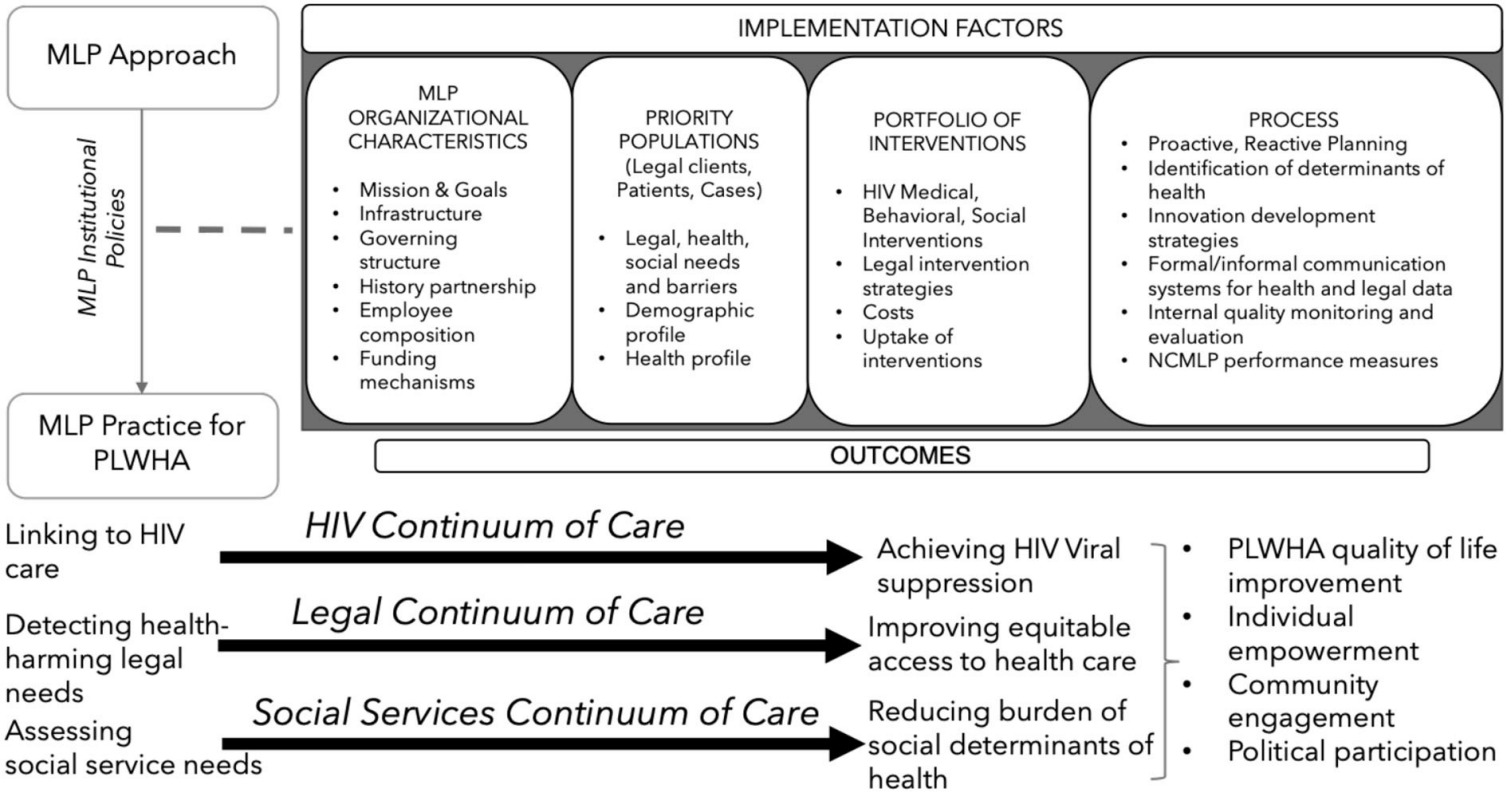
Theoretical Framework. In building the structure used to present this theoretical framework for integrated continua of care, investigators referenced McRobie et al. (41).

This is one of the first studies to examine MLPs as a structural intervention to address disparities in HIV/AIDS treatment and outcomes through continuums. This study identified three synergistic care continua – HIV care continuum, legal continuum, and social services continuum–within MLPs. The integration of services is increasingly recognized as a means to develop more effective models of care and improve patient outcomes. This integrated approach to care, including legal and social services, is imperative to address the HIV/AIDS epidemic. Drawing from the HIV Implementation Science Model, the investigators illustrate the interaction among the 3 care continua and potential outcomes for PLWH (see Figure 1) (41). This collaborative approach requires organization, communication, and cooperation among MLP partners.

### Medical legal partnerships as an HIV structural intervention

Conceptually, MLPs have the capacity to create sustainable, efficient, comprehensive structural changes that minimize barriers to HIV care and treatment and improve health and quality of life outcomes. The effects of these structural-level changes have the potential to reverberate throughout other levels of the ecological model and create population level structural changes that impact communities, organizations and institutions, interpersonal relationships, and individuals (25).

Synergistic care continua approaches are imperative to address the HIV epidemic. Integration of services is increasingly recognized as a means to develop more effective models of care and improve patient outcomes (42, 43). The philosophy behind the HIV continuum of care is that in order to achieve HIV viral suppression and improve the quality of life for PLWH, the person must first know their HIV status and, if positive, access and engage in HIV treatment and care services. The legal continuum of care follows a similar philosophy. In order to provide comprehensive HIV care we must follow the legal continuum of care for PLWH, as identified in our prior study: we first must identify the health-harming legal needs that preclude access to care in the first place, provide timely linkage to legal support, facilitate engagement in legal services, and achieve resolution of legal issue(s) (44). The approach presented here to addressing health-harming legal needs for PLWH is based on the principles of integrated care and co-location of services in order to reduce redundant, unnecessary costs and streamline services (45).

**Figure 2:**
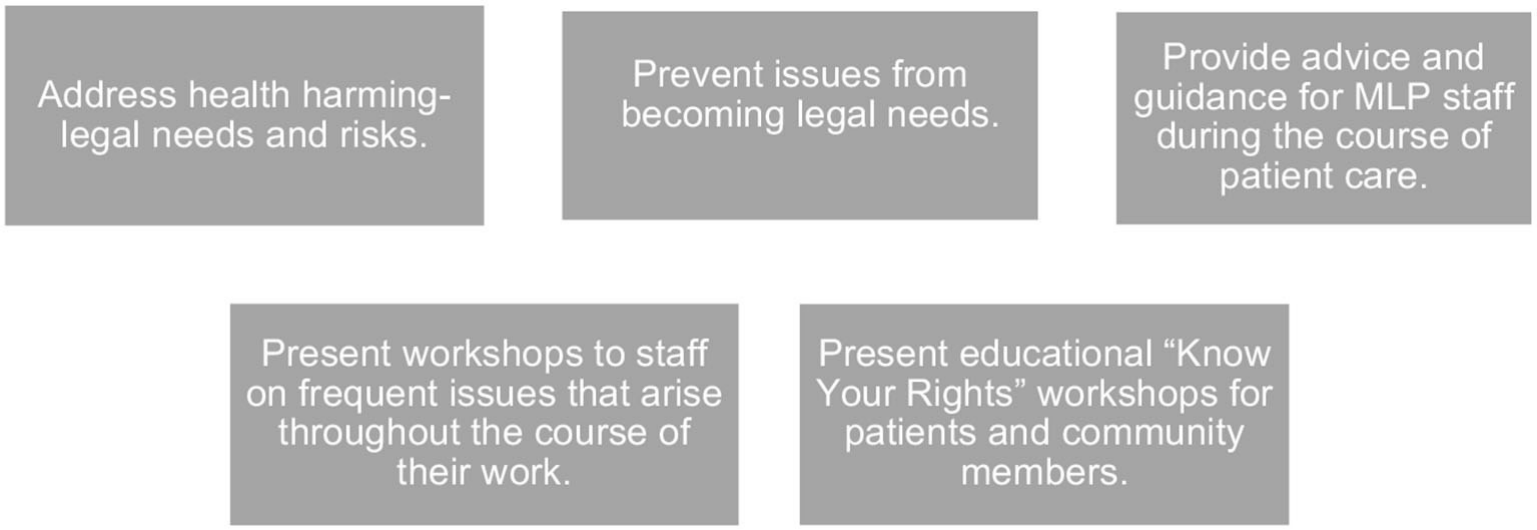
Added value of attorney in health care team.

Our study also documents the importance and added value of the attorney in the health-care team (see Figure 2). Though case managers are highly qualified to coordinate medical care and other services, current professional practice norms – especially relating to the unauthorized practice of law – mean that they cannot substitute for legal advocates in most instances. The integration of legal specialists into the health care system serving PLWH has many significant benefits. First, legal partners are uniquely equipped to address health-harming legal needs, such as those mentioned above. In the absence of an attorney, case managers lack a “hammer” when confronted with unresponsive agencies and decision makers; “messenger matters” and communication from an attorney followed by legal action, if necessary, is often more impactful. Providing workshops to health center staff of common questions, issues, or changes in the law is a critical component of legal service integration. Lastly, attorneys can conduct “Know Your Rights” workshops to educate and empower patients and the community to help them advocate for themselves. (Can you be evicted? How to identify discrimination. Are you eligible for benefits?) Attorneys can also provide advice and guidance to health center staff during the course of patient care (e.g., proper documentation for disability benefits). The inclusion of a legal specialist on the health care team makes it more likely that issues can be identified before they rise to a level that requires legal intervention (e.g., illegal threat of eviction).

One factor hindering the broad adoption of MLPs as a structural HIV intervention is the absence of quantifiable research analyzing the health outcomes and cost-benefit implications of this approach. It is widely accepted that preventive care is a cost-saving measure, addressing health issues before they become complicated and expensive crises. Viewing MLPs through the preventive care lens, it will be important to investigate the costs and benefits for various combinations of integrated services. For example, what is the impact of legal service integration into clinical care vs. the integration of social services into clinical care? How do those models compare to models that integrate all three care continua? Although key informants in this study provided compelling anecdotal support for MLPs, systemic, empirical investigations are needed to assess the impact of various MLP models on health outcomes among diverse patient populations, including PLWH.

### Future research and directions

Given the potential impact of MLPs on HIV care continuum outcomes, more longitudinal studies are needed to assess the potential of MLPs to address health outcomes for PLWH as well as other comorbidities, such as substance use, mental illness, and COVID-19. Research is also needed into communication management, both internally among MLP partners and externally with patients and clients. Training modules should also be developed and evaluated toenable physicians, health care providers, and non-legal MLP staff to identify potential legal issues that impact the health of MLP patients and the role they play in meeting clients legal needs, such as providing medical evidence required to obtain appropriate benefits and employment accommodations. Further, we need to gaining a better understanding of MLP costs and benefits, particularly as new healthcare payment models emerge. One factor hindering the broad adoption of MLPs as a structural HIV intervention is the absence of quantifiable research analyzing the health outcomes and cost-benefit implications of this approach. It is widely accepted that preventive care is a cost-saving measure, addressing health issues before they become complicated and expensive crises. Viewing MLPs through the preventive care lens, it will be important to investigate the costs and benefits for various combinations of integrated services. For example, what is the impact of legal service integration into clinical care vs. the integration of social services into clinical care? How do those models compare to models that integrate all three care continua? Although key informants in this study provided compelling anecdotal support for MLPs, systemic, empirical investigations are needed to assess the impact of various MLP models on health outcomes among diverse patient populations, including PLWH.

### Limitations

First, this study is based on a convenience sample of organizations which provide HIV services in major HIV epidemiological centers around the country. Thus, the findings and their implications may not capture the organizational experiences of MLPs operating in low HIV prevalence geographical locations. Second, although the HIV care continuum model has been well documented through expert panels, the legal continuum is a new concept based on the information gathered during this study. Further research is needed to validate the legal continuum of care and its intersection with the HIV and social services continua of care. Third, one of the major challenges of conducting organizational level research is the plurality of information. As in other forms of research, this analysis is influenced by investigators' professional positions. Fourth, while participants shared information related to patient demographics and characteristics, we didn't collect or retain this information as part of this organizational-level qualitative research. Fifth, the integrated models of care presented have not been evaluated for feasibility and acceptability or efficacy. Theory-guided evaluation models for integrated care approaches are urgently needed.

## Conclusion

The MLP model holds great promise as an effective and innovative structural intervention to address the HIV epidemic domestically and globally. Mitigating SDoH and addressing health harming legal needs through the integrated continua of health, legal, and social services are at the core of MLP effectiveness in improving health outcomes for PLWH. The analysis of MLPs organizational practices provides the opportunity to illustrate the public health importance of the integration of the care continua and the potential to significantly reduce HIV co-morbidities and HIV transmission by removing barriers to successful HIV treatment.

## Data availability statement

The raw de-identified data supporting the conclusions of this article will be made available by the authors, without undue reservation.

## Ethics statement

The studies involving human participants were reviewed and approved by Temple University Institutional Review Board. The patients/participants provided their written informed consent to participate in this study.

## Author contributions

All authors listed have made a substantial, direct, and intellectual contribution to the work and approved it for publication.

## Funding

This study was supported by the United States National Institute of Mental Health (NIMH) grant # R21MH115820 (to OM and MM-L).

## Conflict of interest

The authors declare that the research was conducted in the absence of any commercial or financial relationships that could be construed as a potential conflict of interest.

## Publisher's note

All claims expressed in this article are solely those of the authors and do not necessarily represent those of their affiliated organizations, or those of the publisher, the editors and the reviewers. Any product that may be evaluated in this article, or claim that may be made by its manufacturer, is not guaranteed or endorsed by the publisher.
